# Elevated Serum Levels of Monocyte Chemotactic Protein-1/Chemokine C-C Motif Ligand 2 are Linked to Disease Severity in Patients with Fibromyalgia Syndrome

**DOI:** 10.4274/balkanmedj.galenos.2019.2019.6.47

**Published:** 2019-10-28

**Authors:** Yuan-Chuang Zhao, Ting Hu, Yan Chen, Ke-Tao Du

**Affiliations:** 1 Department of Rehabilitation and Physiotherapy, Guangdong Provincial Corps Hospital of Chinese People’s Armed Police Forces, Guangzhou Medical University, Guang Dong Province, China; 2 Beijing International Travel Healthcare Center, Beijing, China; 3 Department of Rehabilitation, Chenzhou NO.1 People’s Hospital, Chenzhou, Hunan Province, China

**Keywords:** Chemokine CCL2, fibromyalgia, monocyte chemotactic protein-1

## Abstract

**Background:**

Elevated levels of monocyte chemotactic protein-1/chemokine C-C motif ligand 2 have been identified in fibromyalgia patients.

**Aims:**

To examine the potential association among serum levels of monocyte chemotactic protein-1/chemokine C-C motif ligand 2 with disease severity of fibromyalgia.

**Study Design:**

Cross-sectional study.

**Methods:**

Seventy-nine female patients with fibromyalgia and 75 healthy normal controls were included in our study. Serum levels of monocyte chemotactic protein-1/chemokine C-C motif ligand 2 were detected by enzyme-linked immune sorbent assays. The existence of tender points was evaluated based on the standardized manual tender point examination. Pressure pain thresholds at the knees, and bilateral trapezius muscles were measured with an algometer. A visual analog scale and the Revised Fibromyalgia Impact Questionnaire were utilized to assess the degree of pain and functional abilities.

**Results:**

Serum levels of monocyte chemotactic protein-1/chemokine C-C motif ligand 2 were significantly greater in patients with fibromyalgia compared with healthy controls (151.6±31.9 pg/mL vs 103.3±25.2 pg/mL, p<0.001). Patients with severe fibromyalgia had significantly higher serum levels of chemokine C-C motif ligand 2 than patients with mild and moderate fibromyalgia (173.1±21.9 pg/mL vs 151.0.0±35.1 pg/mL, p=0.01). Patients with moderate fibromyalgia revealed markedly augmented serum levels of chemokine C-C motif ligand 2 compared with patients with mild fibromyalgia (151.0±35.1 pg/mL vs 133.3±23.9 pg/mL, p=0.03). Serum levels of chemokine C-C motif ligand 2 were positively associated with tender point scores (r=0.455, p<0.001). In addition, serum levels of chemokine C-C motif ligand 2 were positively associated with pressure pain thresholds in both knees and bilateral trapezius muscles (knees: r=-0.349, p=0.002; trapezius muscles: r=-0.318, p=0.004). Finally, we found elevated serum levels of chemokine C-C motif ligand were also positively associated with the visual analog scale (r=0.368, p=0.001), and the Fibromyalgia Impact Questionnaire score (r=0.401, p<0.001).

**Conclusion:**

Elevated serum levels of monocyte chemotactic protein-1/chemokine C-C motif ligand 2 are linked to disease severity of fibromyalgia. Therapeutic interventions inhibiting monocyte chemotactic protein-1/chemokine C-C motif ligand 2 in fibromyalgia deserve additional studies.

Fibromyalgia (FM) is a complicated chronic disorder that affects nearly 6% of adults and is mainly characterized by widespread pain (1,2). FM is also accompanied by other symptoms in patients, for example, non-restorative sleep, tiredness, poor physical conditioning, diminished cognition, stiffness, depression, and balance problems (3), leading to a significant economic burden because of the high prevalence of loss of work (4).

Even though the causes of FM remain unknown, sensitization of the central nervous system and musculoskeletal dysregulation has been regarded as two vital factors in the advancement of FM (5). This dysregulation may indicate that the reason for the high level of pain is the magnification of sensory inputs from the central nervous system to the musculoskeletal system.

Over the past several years, immunological dysfunction and inflammation have been shown to play essential roles in FM (6). As one type of important inflammatory cytokine, chemokines are comprised of a family of small soluble molecules of around 70 amino acid residues with a molecular weight of 7-12 kDa (7). Chemokine ligands are separated into four families (C, CC, CXC, and CX3C) as per cysteine residue regions, and every ligand has specific effects on numerous cells via the stimulation of ligand-specific receptors (8). Chemokine ligands and receptors are widely expressed throughout organs, tissues, and cells, under physiological as well as pathological conditions (9). They regulate chemotaxis and the stimulation of numerous kinds of populations of leukocytes, and hence, are major controllers of leukocyte traffic (10). Under pathological conditions, chemokines play pivotal roles in broad reactions of inflammatory and immune responses via the chemoattraction of innate plus adaptive immune cells (11).

Monocyte chemotactic protein-1 (MCP-1), also known as chemokine C-C motif ligand 2 (CCL2), is a chemotactic cytokine under the CC chemokine family (12). CCL2/MCP-1 acts as an essential factor for the recruitment and trafficking of mononuclear and immune cells to the sites of inflammation (13) and has pivotal functions in the development of chronic inflammatory syndromes, such as osteoarthritis, atherosclerosis, rheumatoid arthritis, and multiple sclerosis (14,15,16).

Recent studies have suggested the potential role of MCP-1/CCL-2 in FM. High levels of MCP-1/CCL-2 have been detected in patients with FM compared with controls (17). In addition, myoblasts can secrete MCP-1, whereas treatment with MCP-1 stimulates the secretion of IL-1β (17). In a rodent experimental study, MCP-1/CCL2 caused long-lasting mechanical hyperalgesia and induced a state of chronic sensitization to other algogens (18), through stimulation of its binding receptor CCR2. MCP-1/CCL2 is also involved in the initiation of persistent muscle pain after repetitive exposure to stressful stimuli (18). In addition, injection of MCP-1/CCL2 into the gastrocnemius muscle led to a dosage- and time-dependent reduction in the mechanical nociceptive threshold (18).

All the studies mentioned above suggest that MCP-1/CCL-2 plays an essential function in the pathogenesis and advancement of FM. Nevertheless, none of the studies has investigated the relationship among serum levels of MCP-1/CCL-2 and disease severity of FM. Thus, the purpose of the current study was to determine if circulating levels of MCP-1/CCL-2 are correlated with disease severity in patients with FM.

## MATERIALS AND METHODS

### Study patients

From September 2017 to January 2019, 79 patients diagnosed with FM syndrome who satisfied the American College of Rheumatology criteria for FM syndrome were allowed to participate in this study (19). Patients were excluded if they had active inflammatory or autoimmune diseases; unstable medical or psychiatric illness; hemorrhaging or active bleeding; thrombosis or angina pectoris; pregnancy or lactation; heart disease; substance abuse in the last year; lumbar or cervical disk disease; and back or neck injuries within the last six months. All criteria were pre-evaluated and confirmed by our senior doctors before enrollment. Healthy controls comprised 75 healthy women who received regular body examinations during the same period. The collected data also comprised height and weight measurements, which were utilized to calculate body mass index. The current study was approved by the local Institutional Review Board. The ethical number is CZSDYRMYY20170012 and the date is 2017.05.01.Patients provided signed informed consent.

### Definition of tender point score

The existence of tender points was evaluated based on the standardized tender point manual examination (19). The number of tender points was recorded at 18 designated places on the body, and the extent of each tender point was evaluated by the following: 0, no tenderness; 1, mild tenderness (identified response when asked); 2, moderate tenderness (spontaneous response); and 3, severe tenderness (cannot bear and moved away). Hence, the probable number of tender points ranged between 0 and 18, whereas the probable total score ranged between 0 and 54. In our study, tender point scores between 0 and 18 were regarded as mild, whereas scores between 19 and 36 were defined as moderate, and scores between 37 and 54 were regarded as severe. The tender points of all patients were assessed by two experienced independent therapists. The *Kappa* value was recorded for the consistency of the tender point calculation.

### Assessment of pressure pain thresholds

Pressure pain thresholds (PPTs) at the knees and bilateral trapezius muscles (joint and non-joint) were measured using a Wagner Force 10 FDX Algometer (Wagner Instruments, USA). The 1 cm2 rubber algometer probe was available at each site selected by the physician. The pressure was augmented at a rate of 0.50 kgf per second until the presence of pain, which was defined as the PPT. Low PPTs at the knee joint were regarded as indicators of peripheral sensitization, whereas low PPTs at the knee and the trapezius muscles were regarded as markers of central sensitization (20). To acquire the mean PPT for each site, trials were repeated three times. We averaged the PPTs acquired on both sides during all three tests.

### Definition of clinical severity

The clinical assessment of FM was performed using the visual analog scale (21), and the revised version of the Fibromyalgia Impact Questionnaire, the FIQR (22). For the visual analog scale, patients were shown a 10 cm ruler with markings that ranged from no pain to the worst presumable pain on the right. Patients were requested to indicate the point on the ruler that corresponded to their level of pain. Scores were recorded by determining the distance between the 0 cm point and the point indicated by the patient, with scores ranging from 0 to 10. The total score of the FIQR ranges from 0 to 100 and includes three items: functional activity (0-30 points) including nine items, overall impact condition (0-20 points) with two items, and symptomatic severity (0-50 points) containing ten items (22). Higher scores on the FIQR indicated worse status compared with lower scores.

### Laboratory examination

Serum was acquired from the blood obtained by antecubital vein puncture at 8:00 am before breakfast. Venipuncture of all participants was performed by a senior nurse. Following extraction, blood samples for serum isolation were kept for 20 minutes at room temperature. Then, the blood was centrifuged at 1500 g for 10 min. Next, serum samples were aliquoted and kept at -80 °C before measurement. Serum MCP-1/CCL2 concentrations (R&D Systems, MN, USA) were investigated using commercially available kits according to the suppliers' instruction manuals. The detection range was 31.2-2,000 pg/mL. The intra-assay and inter-assay coefficient of variation for MCP-1/CCL2 were 4.9% and 5.9%, respectively. Each sample examination was repeated three times, and the average result was calculated.

### Statistical analysis

Data analysis was performed with Graphpad 6.0 software (USA). Values are given as means ± standard deviations or medians. The Shapiro–Wilk test was utilized to test for normality of data distribution, and Levene’s test was performed for equality of variances. Group comparisons were calculated using a one-way ANOVA for parametric data, or the Kruskal–Wallis test for nonparametric statistics. A Spearman or Pearson analysis was conducted to test the correlation of CCL2 levels with other parameters. A two-tailed test was done, and p<0.05 was considered statistically significant. The statistical power (1-β) was computed using IBM SPSS 21.0 software. The result was calculated depending on the achieved data of various mean serum levels of MCP-1/CCL2, standard error, and enrolled numbers of patients in each group. Statistical power was considered strong when >0.8. The formula is shown below (23):

$1-\;\beta =\varphi (z-z1-\alpha /2)\;+\varphi (-z-z1-\alpha /2),z\;=\;(\mu A-\mu B)\;/\sigma (\frac{1}{n_{A}}\;+\;\frac{1}{n_{B}})$

## RESULTS

### Demographic data

**Figure 1:**
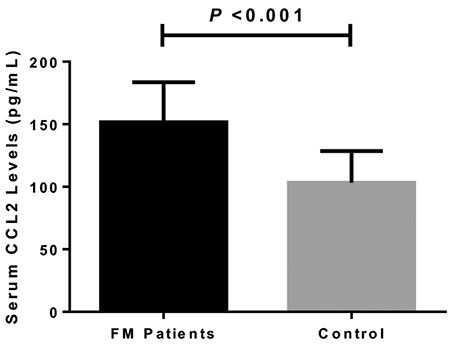
Comparison of serum levels of CCL2 among patients with FM and controls. CCL2: chemokine C-C motif ligand 2; FM: fibromyalgia

The statistical power was 0.97 after quantification. Demographic data of all subjects enrolled in the current study are presented in Table 1. The mean age of females with FM was 42.4±11.7 years, ranging from 22 to 65 years, and the average age of healthy females was 41.9±11.5 years. There were no substantial differences in age between both groups (p=0.178). Also, the difference in body mass index between females with FM and healthy controls did not reach significance (23.2±2.0 kg/m^2^ vs 22.8±1.9 kg/m^2^, p=0.084). Serum concentrations of MCP-1/CCL2 were significantly greater in patients with FM compared with healthy individuals (151.6±31.9 pg/mL vs 103.3±25.2 pg/mL, p<0.001) (Table 1; Figure 1).

### Correlation of serum levels of MCP-1/CCL2 with tender point score

**Figure 2:**
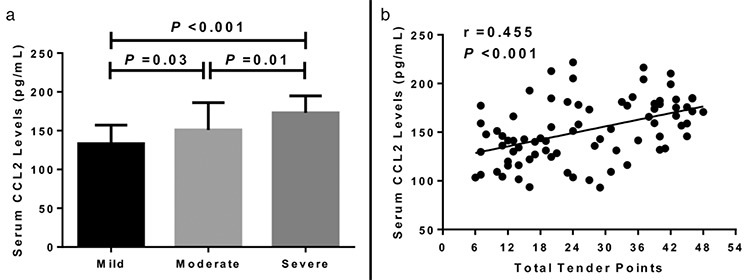
a, b. Assessment of serum levels of CCL2 among patients with FM with different disease extent (a). Relationship of serum levels of CCL2 with total tender points in patients with FM (b). CCL2: chemokine C-C motif ligand 2; FM: fibromyalgia

The *Kappa* value was 0.84 after assessment of tender point scores. According to the classification of disease severity based on the total point scores, 27 patients were defined as mild, whereas 28 were defined as moderate, and 24 were defined as severe. Serum levels of CCL2 were suggestively greater in patients with moderate FM compared with females with mild FM (151.0±35.1 pg/mL vs 133.3±23.9 pg/mL, p=0.03). Patients with severe FM had markedly elevated serum levels of CCL2 than patients with moderate FM (173.1±21.9 pg/mL vs 151.0.0±35.1 pg/mL, p=0.01) and patients with mild FM (173.1±21.9 pg/mL vs 133.3±23.9 pg/mL, p<0.001) (Figure 2a). Last, we found that serum levels of CCL2 were suggestively and positively related to total tender points (r=0.455, p<0.001) (Figure 2b).

### Correlation of serum levels of MCP-1/CCL2 with PPTs

**Figure 3:**
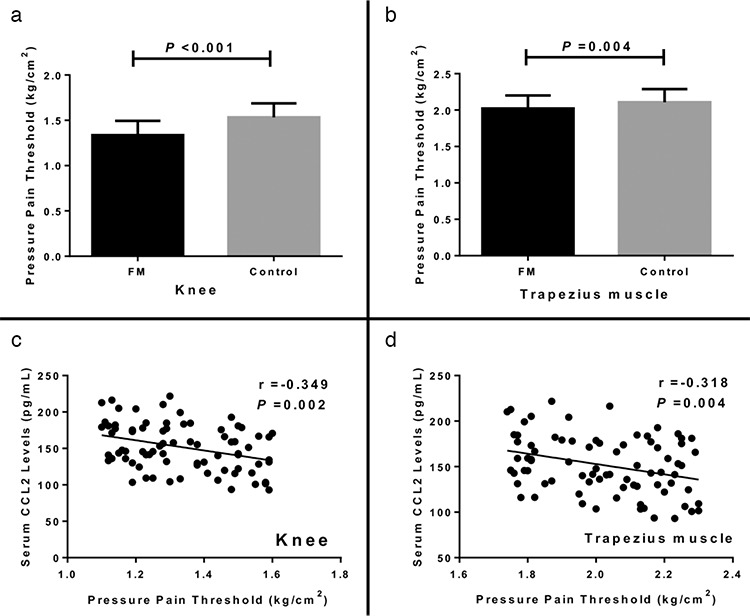
a-d. Assessment of average knee pressure pain threshold between patients with FM and controls (a). Comparison of average trapezius muscle pressure pain threshold between patients with FM and controls (b). Relationship of serum levels of CCL2 with average knee pressure pain threshold in patients with FM (c). Relationship of serum levels of CCL2 with trapezius muscle knee pressure pain threshold in patients with FM (d). CCL2: chemokine C-C motif ligand 2; FM: fibromyalgia

We explored the systemic levels of MCP-1/CCL2 on pain sensitization further by testing the pressure pain on the knee and the trapezius muscle as mentioned above. We found that the average PPTs of the knee and the trapezius muscles were statistically less in patients with FM compared with those in controls (average knee PPT: 1.34±0.16 kg/cm^2^ vs 1.53±0.15 kg/cm^2^, p<0.001; average trapezius muscle PPT: 2.02±0.18 kg/cm^2^ vs 2.10±0.18 kg/cm^2^, p=0.004) (Figure 3a, 3b). In addition, we found elevated serum levels of MCP-1/CCL2 were related to average PPTs of the knee and trapezius muscles (knee: r=-0.349, p=0.002; trapezius muscles: r=-0.318, p=0.004) (Figure 3c, 3d).

### Correlation of serum levels of MCP-1/CCL2 with clinical severity

**Figure 4:**
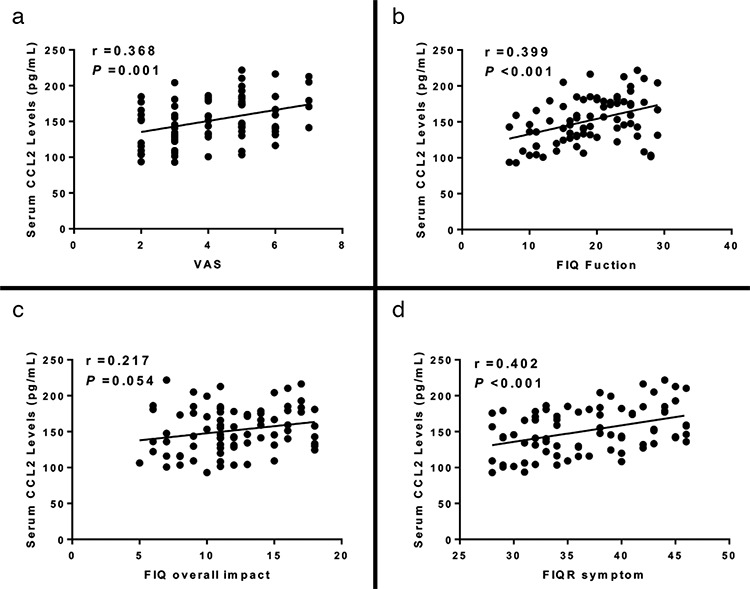
a-d. Relationship of serum levels of CCL2 with the VAS score in patients with FM (a). Relationship of serum levels of CCL2 with FIQ function score in patients with FM (b). Relationship of serum levels of CCL2 with FIQ overall impact score in patients with FM (c). Relationship of serum levels of CCL2 with FIQ symptom score in patients with FM (d). CCL2: chemokine C-C motif ligand 2; FM: fibromyalgia; FIQ: Fibromyalgia Impact Questionnaire; VAS: visual analog scale

We last examined the relationship between serum levels of MCP-1/CCL2 with clinical severity distinguished by the visual analog scale score and the FIQ scale. We found that serum MCP-1/CCL2 intensities were positively related to the visual analog scale score (r=0.368, p=0.001) (Figure 4a). In addition, serum MCP-1/CCL2 intensities were also positively related to the revised FIQ function (r=0.399, p<0.001) (Figure 4b) and symptom score (r=0.401, p<0.001) (Figure 4d). Although the differences between serum levels of CCL2 and the FIQ overall impact score did not achieve significance, a slight correlation still existed (r=0.217, p=0.054) (Figure 4c).

## DISCUSSION

The current investigation seeks to inspect the relationship of serum levels of MCP-1/CCL2 with disease severity in females with FM syndrome. We observed that serum concentrations of MCP-1/CCL2 were positively associated with higher tender point scores, lower PPTs, and clinical severity in females with FM. Our findings, along with other previous studies, indicated the critical involvement of MCP-1/CCL2 in the FM pathophysiological process.

In recent years, the examination of particular and detectable biomarkers that might help in accurately recognizing susceptible individuals, verifying disease diagnosis and expediting treatment, has been regarded as a novel method in FM research (24). The development of mainly relevant potential biomarkers has confirmed neurological research where small molecules are vital in neurochemical metabolism and play crucial roles as neurotransmitters and signaling modulators (25).

Advancements in basic and clinical research over the years have validated the idea that FM is not only a central sensitivity disorder related to abnormal pain processing but a broader condition that requires a comprehensive methodology. This method also must comprise a systemic approach that considers the substantial influence of the inflammatory reaction (6). It is now generally anticipated that an array of inflammatory markers, connected to systems biology approaches, will transpire with well-defined phenotypes, increasing the understanding of the pathophysiological process of FM (26). In the previous studys, a few inflammatory biomarkers in peripheral blood have been documented in FM, possibly revealing new insight into inflammation in FM (27,28). The activated pain-related nervous system that characterizes FM is dependent on modulation partially by cytokines and chemokines (29).

In the current study, we first observed that serum levels of MCP-1/CCL2 were suggestively greater in patients with FM compared with controls, which is consistent with the previous study (17). In another study, plasma levels of MCP-1/CCL2 were also elevated in women exposed to continued psychosocial stress (30). Pain is the main indication, and both allodynia and hyperalgesia are usual indications in FM. We also found that serum levels of MCP-1/CCL2 were positively related to the tender point score, the visual analog scale score, and the FIQ score. A previous study found that MCP-1/CCL2 and its respective receptor CCR2 are both upregulated in numerous subpopulations of sensory neurons (31). Stimulation of CCR2 by MCP-1/CCL2 causes membrane depolarization, activates action potentials, and sensitizes nociceptors via transactivation of transient receptor potential channels, TRPA1, and TRPV1 (31). All these studies indicate that MCP-1/CCL2 participates in pain in FM.

This study has certain limitations. First, our study only included female patients with FM; investigation of MCP-1/CCL2 levels in male patients would help to obtain a more accurate conclusion. Second, the current study only included people from China. Therefore, the findings might not be directly valid to individuals of other ethnicities. Third, only serum levels of MCP-1/CCL2 were examined. Examination of other potential chemokines might offer additional vital information. Finally and importantly, the current study was designated as a cross-sectional study. Consequently, no inferences concerning cause and effect associations can be made. Hence, prospective longitudinal studies are needed to chart disease progression and describe the potential function of MCP-1/CCL2 in FM.

Collectively, we showed suggestively elevated systemic expression of MCP-1/CCL2 and demonstrated a significant positive relationship with the extent of disease severity in Chinese females with FM. Our findings might help to improve the current understanding of the function of MCP-1/CCL2 in the pathogenesis of FM.

## Figures and Tables

**Table 1:**
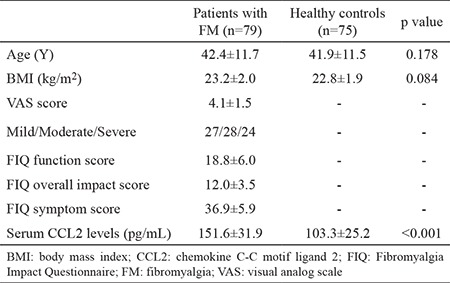
Demographic characteristics of patients
